# Stress Recovery of Campus Street Trees as Visual Stimuli on Graduate Students in Autumn

**DOI:** 10.3390/ijerph17010148

**Published:** 2019-12-24

**Authors:** Li-Na Guo, Ren-Lin Zhao, Ai-Hua Ren, Li-Xin Niu, Yan-Long Zhang

**Affiliations:** College of Landscape Architecture and Arts, Northwest A&F University, Xianyang 712100, China; guolina0811@163.com (L.-N.G.); zhaorenlin@nwafu.edu.cn (R.-L.Z.); aihua.ren@foxmail.com (A.-H.R.)

**Keywords:** campus street tree, graduate student, stress recovery, psycho-physiological indicator

## Abstract

Human stress recovery response to landscapes is under discussion in Chinese settings. The present study aimed to clarify the stress recovery effects of campus street trees on graduate students in autumn. A total of 150 participants (23.75 ± 1.01 years old) completed the Trier Social Stress Test (TSST) and were then randomly assigned to view one of five virtual environments, including the street trees *Sophora japonica*, *Ginkgo biloba*, *Platanus acerifolia*, *Koelreuteria paniculata*, and the indoor environment (control). Physiological responses were measured by R-R interval and electroencephalography (EEG). Psychological responses were examined through the state version of the State-Trait Anxiety Inventory (STAI-S) and the Perceived Restorativeness Scale (PRS). Results showed that R-R intervals significantly increased while viewing all street trees. Both alpha and beta brainwave activities while viewing *S. japonica* and *G. biloba* were remarkably higher than those while viewing *P. acerifolia* and *K. paniculata*. The STAI-S scores significantly decreased, and the positive PRS scores were registered after viewing street trees. We concluded that a brief virtual visual experience of campus street trees in autumn has stress recovery effects on graduate students, and the different levels of stress recovery are associated with different types of street trees.

## 1. Introduction

Stress is a growing public health concern in modern times. A survey in 2018 showed that 41% of graduate students score in the moderate–severe range for anxiety, and 39% have signs of moderate–severe depression [1]. Many of these cases are related to academic, financial, or employment pressures [2], so a clear understanding of intervention to relieve stress is needed to improve students’ health [3]. Increasing evidence suggests that exposure to natural environments or urban green environments have positive effects on stress relief and well-being [4,5,6,7]. Indirect interaction with nature can increase relaxation, such as viewing nature through windows [8,9]. Early studies on preferences or effects of plants intentionally avoided colors other than green to remove distractions [10]. Some researchers have examined flowering plant color preferences. Behe, B. et al. found that red and lavender geranium flowers were preferred over white and pink ones [11]. Green-yellow and bright green foliage developed positive feelings and calmness [12], and yellow flowered plants can be used to create pleasant places [13]. Recent attention has been paid to the role of campus landscapes in reducing stress levels of students [14,15]. However, there is a lack of research-based evidence concerning stress recovery effects of campus street trees on people, especially of graduate students, for the landscapes in autumn. The present study was thus designed to provide such evidence.

### 1.1. Green Campus Landscapes and Health

Green campus landscapes have a close association with the improvement in students’ physical and psychological health [16,17]. Researchers have found that exposure to green spaces is beneficial for students to relieve stress [15,18], promote attention restoration [19], enhance cognitive abilities [20], and improve academic performance [21]. Direct contact with nature is not necessary to facilitate restoration. Viewing nature through windows can help students reduce pressure and mental fatigue [10]. Even viewing images of nature can provide students with effective restoration [22]. These studies suggest that students positively respond to green campus landscapes. The street tree landscapes on campus are relatively accessible green spaces in the busy daily life of graduate students. However, such studies are severely limited.

### 1.2. Street Trees and Health

The primary purpose of street trees has transitioned from an aesthetic role to one that includes ecological service function over the course of the last 30 years [23]. Recent evidence shows a positive correlation between street trees and well-being [24,25]. The empirical research has suggested that streetscape greenery of neighborhoods promote adults’ health through decreased stress and increased social cohesion [26]. The quantity and quality of streetscape in one’s neighborhood is related to self-reported health [27]. In a cross-sectional study conducted in London, the urban street tree density is inversely associated with antidepressant prescription rates [28]. The amount or density of street trees is tested as a positive predictor of stress recovery [29]. The findings demonstrate the positive benefits of green street trees in neighborhood or urban environments.

### 1.3. Overview of the Study

The objective of this study was to examine the stress recovery effects of campus street trees on Chinese graduate students in autumn. In the experiment, we used the Trier Social Stress Test (TSST) to induce acute stress and provided students with the scene experience in virtual visual stimulation. We then quantitatively analyzed the physiological and psychological data. The key research questions are: (1) If a brief virtual visual experience of street trees has stress recovery effects. (2) If there are differences in the relaxing effects among street trees. When we fully understand students’ responses to street trees, landscape designers and administrators could have a theoretical basis for campus streetscapes design and renovation.

## 2. Materials and Methods

### 2.1. The Participants

The study was conducted in accordance with the ethics rules of Northwest A&F University, and informed consent was obtained from all participants. We publicized the experiment through posters and announcements and established a social network discussion group to reach as many potential participants as possible. Altogether, we recruited 150 graduate students (23.75 ± 1.01; mean ± SD) from a wide range of disciplines (12 colleges). Basic information was collected at the time of appointments, such as age, gender, and personal health information. Individuals were excluded who had a history of heart disease (e.g., arrhythmia), emotional disorders (e.g., depression), post-traumatic stress disorder, or 3D vertigo. The participants were randomly divided into five groups. Each group with thirty participants (50% male, 50% female) was arranged to watch one of the five scenes.

### 2.2. The Environmental Settings

The experiment was performed in Northwest A&F University (34.27° N, 108.08° E), China, where 28 main roads were distributed on campus. We took several steps to limit the physical characteristics of the samples. First, we selected campus roads with the same road section forms, widths, and pavement materials. We then excluded samples of adjacent buildings on both sides of the roads. Finally, we focused on the street trees in the form of linear planting with a single-layer arbor structure. Through this process, four kinds of high-frequency and representative streetscapes with *Sophora japonica*, *Ginkgo biloba*, *Platanus acerifolia*, and *Koelreuteria paniculata* planted, respectively, were determined (Table 1).

**Figure 1:**
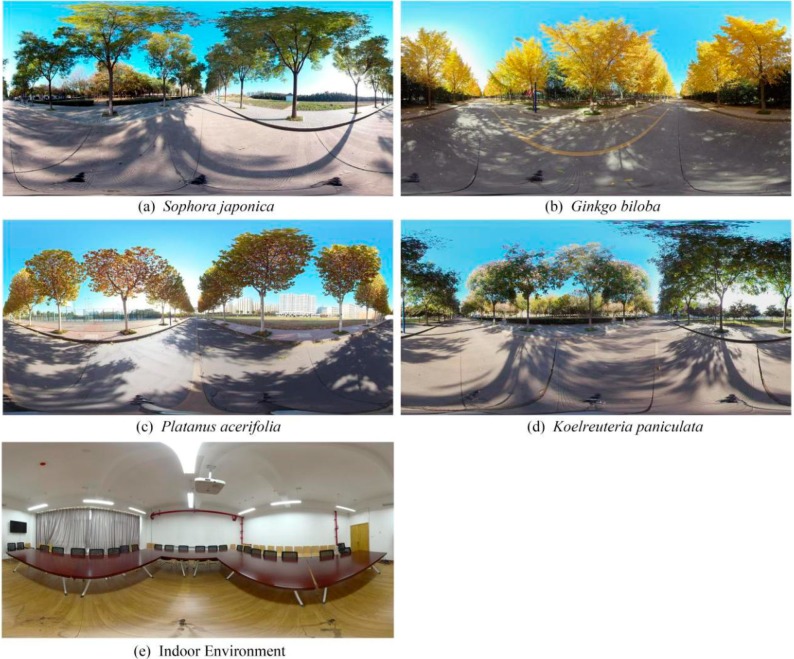
The panorama of experimental scenes in the VR model. (**a**) *Sophora japonica*, (**b**) *Ginkgo biloba*, (**c**) *Platanus acerifolia*, (**d**) *Koelreuteria paniculata*, (**e**) Indoor environment.

Real environments are dynamic settings along with interference factors, such as noise, passersby, traffic flow, and so on. We can eliminate some interference factors by simulating a natural environment. Compared to static simulation (e.g., photographs or slides), virtual environments provide a more immersive situation [30]. Virtual environments of our study were presented using a panoramic camera (Insat360 Pro; Arashi Vision Co., Ltd, ShenZhen, China) and a pair of head-mounted virtual reality (VR) glasses (Pico Goblin A7210; Goertek Inc., Weifang, China). The participants viewed scenes in VR glasses contributing to improve visual realism and offer an immersive experience [31]. The 360-degree panoramic photographs were taken between 10:00 and 16:00 on sunny days without strong winds in the middle of autumn (October 2018). The camera was placed on the center line of the road, and the viewpoint height was set to 1.60 m [32]. The photographs contained no pedestrians or vehicles. The indoor environment that was relatively closed without green views was also taken in a panoramic view. All panoramic photographs had a resolution of 7680 × 3840 pixels (Figure 1).

### 2.3. Stress Induction

The TSST is a well-validated and widely used stressor for inducing acute stress in lab settings [33,34]. It has been proven to activate the hypothalamic-pituitary-adrenocortical axis (HPA) along with the corresponding cardiovascular responses. Yang, J. demonstrated that the TSST is a helpful protocol with good applicability in Chinese college students and could be used to conduct the relevant research on stress [35].

The TSST comprised of two tasks, i.e., impromptu speech and oral calculation. It was performed by two examiners face-to-face with time constraints. First, participants delivered a three-minute speech describing why they would be good candidates for an ideal job. Second, participants were asked to sequentially subtract the number 13 from 1022 for three minutes. They reported their answers quickly and accurately and were asked to restart from 1022 if a mistake was made.

### 2.4. Measures of Stress Levels

#### 2.4.1. Physiological Indicators

R-R interval: This study used a heart rate monitor (Polar V800; Polar Electro Oy., Kempele, Finland) to record the R-R interval (the peak-to-peak interval of heartbeats) [36]. The R-R interval carries significant information about the control of heart rate through the autonomic nervous system and is considered as a more reliable stress assessment indicator [37]. The R-R interval is shorter when the heart rate is faster, reflecting increased stress responses.

EEG: The EEG, which is a recording of fluctuating electrical brainwave activities from the Fp1 position above the eye, was measured using a portable MindWave-EEG headset (NeuroSky; Beijing Vision Technology Co., Ltd, Beijing, China). This headset consists of four essential parts: (i) a headband; (ii) an ear-clip; (iii) a sensor arm containing the EEG electrode; and (iv) a Bluetooth USB. The instrument measures the raw signal, power spectrum (delta, theta, low alpha, high alpha, low beta, high beta, low gamma, and high gamma), mediation level, and attention level. Ahmad, H. et al. used the EEG headset to investigate the relaxing effects of gardening activities on older adults by low alpha, high alpha, low beta and high beta indexes [38], and the relaxing effects of walking in the forest on young adults by high alpha and high beta indexes [39]. Alpha and beta waves have been previously used to record and analyze physiological responses to stress [40,41]. Low alpha, high alpha, low beta, and high beta were selected as evaluation indicators in this experiment (Table 2).

#### 2.4.2. Psychological Indicators

State Anxiety: Anxiety is an emotional experience under stress. To assess subjective stress, the state version of the State-Trait Anxiety Inventory (STAI-S) was used to assess short-term emotional states, such as stress or anxiety [42,43]. The STAI-S is composed of 20 questions (e.g., I am nervous; I am relaxed.) with a 4-point scale from 1 = Not at all to 4 = Completely. Higher scores predict higher anxiety levels.

Restorativeness: The Perceived Restorativeness Scale (PRS) is a commonly used evaluation tool in restorative environment studies [44,45]. The questionnaire consists of 20 items scored on a five-point scale from −2 (nothing) to 2 (extremely) [46]. The total PRS scores indicate the perceived restorativeness of subjects.

### 2.5. The Procedure

**Figure 2:**
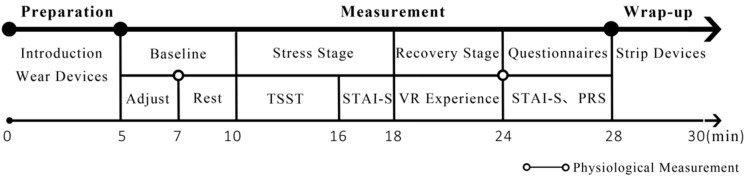
The procedure of the experiment.

The whole experiment was conducted in a quiet lab away from the teaching area to avoid interference from other factors, such as noise. As shown in Figure 2, the test was carried out according to the following protocols:

1. Preparation: The experiment was performed individually. After arriving in the lab, the participant was told the procedure and guided to wear a heart rate monitor and an EEG headband, while sitting in a chair.
2. Baseline: The participant had two minutes to adjust the devices. Then, he or she was asked to relax for three minutes while recording R-R intervals and EEG.
3. Stress stage: The participant took the TSST to induce acute stress for six minutes, and then filled in the STAI-S.
4. Recovery stage: The participant was randomly assigned to view one of the five scenes for six minutes while sitting in the chair. Through the VR glasses, the participant was immersed in the environment of a 360-degree photo by simply moving their head to explore the surroundings. Then, they also did both the STAI-S and the PRS questionnaires.

R-R intervals and ECG were recorded continually while participants experienced the TSST and viewed the scenes. The experiment lasted for about thirty minutes.

### 2.6. Data Analysis

The experimental data were analyzed by SPSS19.0 (IBM SPSS Statistics, Armonk, NY, USA). One-way analysis of variances (ANOVA) was used to evaluate the significance of differences in psycho-physiological data for all groups. Paired sample *t*-tests were used to compare the means of psycho-physiological data before and after the stress stage and the recovery stage. The effect size was measured using Cohen’s *d* with an effect size of 0.20 being considered as small, 0.50 as moderate, and 0.80 or greater as large [47].

## 3. Results

### 3.1. Tests for the Baseline Levels and the Validity of the Stressor

One-way analysis of variance showed that there were no significant differences in the baseline levels of R-R intervals (F = 0.565, *p* = 0.689), the low alpha mean values (F = 0.332, *p* = 0.802), the high alpha mean values (F = 1.643, *p* = 0.179), the low beta mean values (F = 1.523, *p* = 0.218), and the high alpha mean values (F = 2.711, *p* = 0.053).

We conducted paired *t*-tests to examine the mean values of physiological data from the baseline to the time immediately following the TSST for all participants. As shown in Table 3, the TSST provoked a significant decrease in R-R intervals (*p* < 0.001, Cohen’s *d* = 1.029), the low alpha mean values (*p* < 0.01, *d* = 0.345) and the high alpha mean values (*p* < 0.001, *d* = 0.679). There was a significant increase in the low beta mean values (*p* < 0.001, *d* = 1.117) and the high beta mean values (*p* < 0.001, *d* = 1.342). These data indicated that the TSST produced effective stress stimulation for graduate students.

### 3.2. Tests for Physiological Effects of Different Scenes

#### 3.2.1. R-R Interval

**Figure 3:**
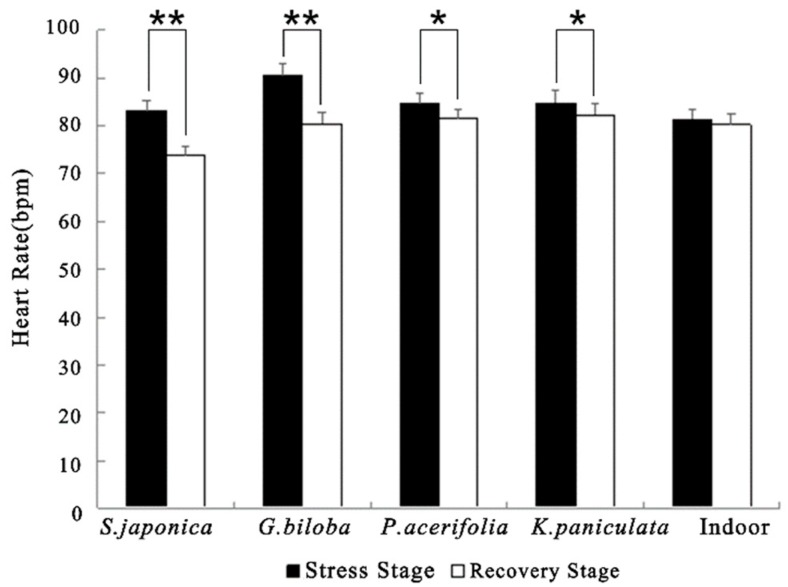
Comparison of mean values of heart rate in different scenes. *N* = 30, mean ± SEM. Note: * *p* < 0.05, ** *p* < 0.01, paired sample *t*-test.

From the stress stage to the recovery stage, the R-R interval values of graduate students in all groups showed an upward trend. One-way analysis of variance displayed significant differences in changes of R-R intervals by the five scenes (F = 3.17, *p* < 0.05). As indicated in Table 4, the R-R intervals increased significantly while viewing street trees *S. japonica* (*p* < 0.001, *d* = 0.918), *G. biloba* (*p* < 0.001, *d* = 0.696), *P. acerifolia* (*p* < 0.01, *d* = 0.627), and *K. paniculata* (*p* < 0.05, *d* = 0.407), comparing to the indoor environment (*p* > 0.05, *d* = 0.150). Furthermore, the heart rate values were significantly reduced while viewing street trees (for all *p* < 0.01, *p* < 0.05; Figure 3).

#### 3.2.2. EEG

**Figure 4:**
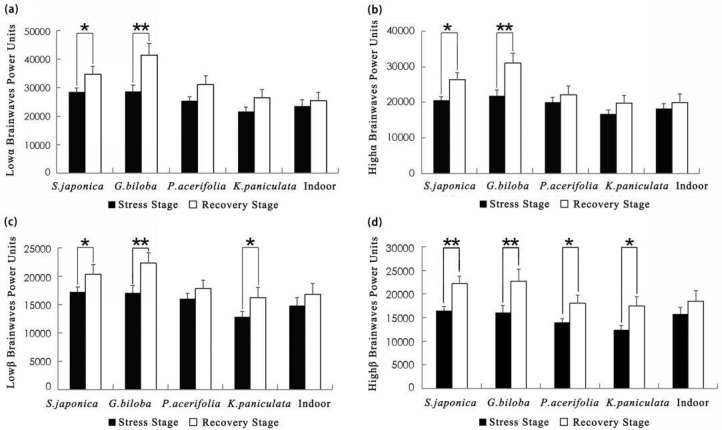
Comparison of mean values of (**a**) low alpha, (**b**) high alpha, (**c**) low beta, and (**d**) high beta brainwaves in different scenes. *N* = 30, mean ± SEM. * *p* < 0.05, ** *p* < 0.01, paired sample *t*-test.

The mean values of low and high alpha, low and high beta increased during the recovery stage, comparing to the stress stage (Figure 4). Paired *t*-tests revealed that the low alpha mean values of viewing street trees *G. biloba* increased the most (*p* < 0.01), *S. japonica* took second place (*p* < 0.05), *P. acerifolia*, *K. paniculata*, and the indoor environment showed no significant difference. The high alpha mean values exhibited a similar trend of increase in five scenes (*G. biloba*: *p* < 0.01; *S. japonica*: *p* < 0.05). Moreover, the low beta mean values showed significant differences while viewing *G. biloba* (*p* < 0.01), *S. japonica* (*p* < 0.05), and *K. paniculata* (*p* < 0.05). The high beta mean values showed significant differences while viewing all street trees (for all *p* < 0.01, *p* < 0.05). There was no significant difference observed in the indoor environment.

### 3.3. Tests for Psychological Effects of Different Scenes

#### 3.3.1. State Anxiety

**Figure 5:**
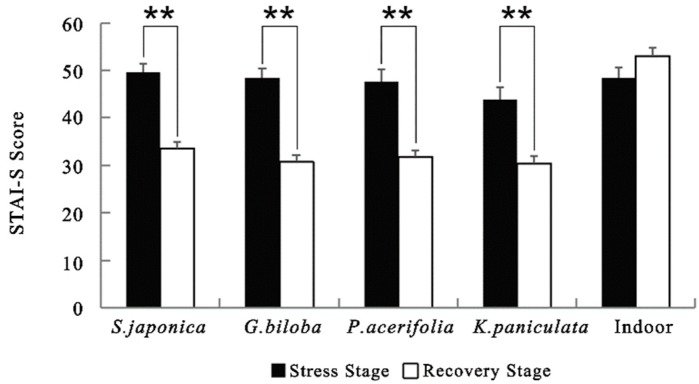
Comparison of STAI-S scores in different scenes. *N* = 30, mean ± SEM. ** *p* < 0.01, paired sample *t*-test.

Paired *t*-tests showed that the STAI-S scores significantly decreased after viewing street trees (for all *p* < 0.01; Figure 5). No significant difference was found in levels of anxiety relief in the indoor environment. The STAI-S scores after viewing street trees *G. biloba* (17.72 ± 2.02) decreased the most, followed by *S. japonica* (15.97 ± 2.05), *P. acerifolia* (15.79 ± 2.53) and *K. paniculata* (13.65 ± 2.29).

#### 3.3.2. Restorativeness

**Figure 6:**
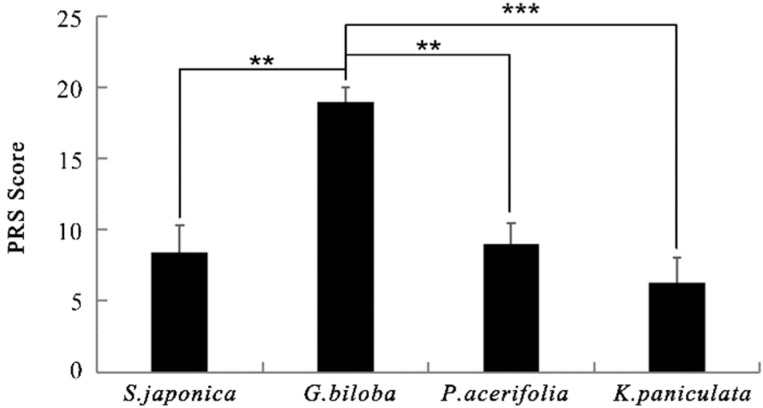
Perceived Restorativeness Scale (PRS) values of different street trees. *N* = 30, mean ± SEM. ** *p* < 0.01, *** *p* < 0.001, one-way ANOVA.

The overall PRS values of four kinds of street trees were positive (Figure 6). One-way analysis of variance showed significant differences in the four scenes (F = 4.76, *p* < 0.01). A Tukey post-hoc test revealed that street trees *G. biloba* were rated high in perceived restorativeness, comparing to the other three street trees (for all *p* < 0.01). The restorative potential was substantially higher in *G. biloba* (18.960 ± 2.040) than that in *S. japonica* (8.417 ± 2.915), *P. acerifolia* (9.000 ± 2.482), and *K. paniculata* (6.267 ± 2.787).

## 4. Discussion

The results identified that campus street trees of autumn in virtual visual stimulation-induced physiological relaxation. R-R intervals markedly increased while viewing street trees after the TSST, and the values indicated that *G. biloba* and *S. japonica* were more effective in stress reduction than *P. acerifolia* and *K. paniculata*. For example, the mean increase in R-R intervals of *G. biloba* was nearly twice as much as *P. acerifolia* (0.090 compared to 0.051), and *S. japonica* was also approximately twice more than *K. paniculata* (0.074 compared to 0.044). In the present study, the low and high alpha, the low and high beta brainwave activities were higher than those during the TSST. The low alpha and high alpha brainwaves were significantly raised while viewing *G. biloba* and *S. japonica*. Low alpha amplitude is related to a sense of security, and high alpha amplitude is related to a feeling of wakeful relaxation [48]. It has also been reported that high alpha is correlated with better academic performance [49]. Alpha waves are closely linked to memory, creativity, perceptual processing, and academic performance, which seem to be in a good state in terms of learning and attention [48,50,51,52]. The current findings suggest that alpha waves increase with feelings of relaxed but wakeful during street trees stimulation. Similarly, the low and high beta waves were significantly higher while viewing *G. biloba* and *S. japonica*. Beta waves occur when the individual is probably more active, alert, and attentive [38,39]. Beta waves are also correlated with task performance [53]. These results agreed with previous studies investigating the restorative effects of plants on EEG [54]. Thus, we concluded that an increase in alpha and beta brainwaves is correlated with mental relaxation and attentiveness.

According to psychological questionnaires, the STAI-S scores were significantly lower after viewing street trees, which demonstrated that the students’ anxiety levels effectively decreased. The total PRS scores of different street trees presented positive results, and *G. biloba* showed more potentially restorativeness. Both psychological responses indicated that viewing street trees could lead to mental stress recovery. The above findings are consistent with previous studies on the psychological benefits of viewing plants [38,55]. Mental health in modern times is considered as an essential component of the overall public health, and the psychological benefits of accessible street trees are expected to play a crucial role in the promotion of human health.

From physiological and psychological perspectives, these results clearly indicated that street trees *G. biloba* and *S. japonica* were more effective in eliciting stress recovery than *P. acerifolia* and *K. paniculata*. The results may be potentially related to visual color responses. Green leaves of *S. japonica* and golden yellow leaves of *G. biloba* evoke positive psycho-physiological responses. People’s feelings change with seasonal changes, which might be a cue to these responses. Green might indicate a healthy and vitality plant, and golden yellow might elicit feelings of warmth, brightness, and comfortableness in the gradually cold autumn. High wavelength colors (e.g., yellow) are more exciting and arousing when measured with physiological measures [56]. Our findings were consistent with previous research, which showed that green plants produced more positive attitudes, and yellow may increase happiness, calmness, and restorative potential [13,57]. The relaxing effects of *P. acerifolia* with yellowing leaves were not as significant as *G. biloba*. The reason may be the difference of color saturation that the leaves of *G. biloba* were golden yellow, while *P. acerifolia* leaves were yellow-brown. It has been stated that preference is more strongly associated with saturation than hue [56]. *K. paniculata*’s role was weaker possibly because dead branches and dead leaves affected visual perception. Its papery seed capsules in multiple colors (e.g., orange to pink and reddish-brown) might cause visual confusion. Additionally, different tree forms, such as tree canopy, trunk size, and shape, might exhibit variable influences on human beings. Research has shown that that people have preferences for trees with larger or spreading canopies [58,59]. In our study, wider canopies of *S. japonica* might elicit more positive emotions than conical, broadly ovoid, and dome-shaped canopies.

This was the first study to take a randomized controlled experiment to examine the beneficial physiological and psychological effects of campus street trees in virtual visual stimulation on graduate students’ stress recovery in autumn. We discussed the implications of these findings here. First, the positive responses of viewing street trees suggest that it may be a simple and attainable method for students to reduce stress and improve health physically and mentally. Campus street trees could serve more functions as health benefits, not just aesthetic roles. Compared to some stress-relieving interventions (e.g., mental health counseling by experts, emotional management courses), planting street trees on campus roads is a low-cost intervention that may generate long-term impacts on generations of students. Therefore, designers should pay more attention to the construction of street trees landscapes and the selection of street tree species. Deciduous trees are proposed in campus design to create seasonal color changes. Green plants can be used in places where easiness and high concentration are required. Trees with yellow leaves in autumn, such as *G. biloba* of higher saturation colors, can be used more to make a place pleasant. Further studies are needed to obtain more detailed design recommendations for landscape designers. Second, landscape management is important to maintain the favorable environment quality of the campus. For example, in autumn, the dead branches and dead leaves of *K. paniculata* could be cleared away by the garden workers in time.

However, this study had several limitations. First, the results cannot be extrapolated to other street trees species, because only four kinds of street trees were selected in this study. Further studies based on a larger sample size, including other various street trees species, are required. Second, to further perceive the differences in stress recovery across different groups of people on campus, subsequent studies could include more diverse occupational populations, such as teachers and administrators. Third, we included a 6-min visual experience of street trees in the study design. Future research should examine whether a longer landscape experience would have a more obvious effect on stress recovery.

In the present study, we used panoramic photographs through VR glasses as visual stimulation. Although this method eliminates distracting factors such as passersby and provides a more immersive situation, it is unclear whether a simulated environment will produce the same effect as a real environment. This is worthy of further research. In addition, a more immersive environment through intelligent media technology in future studies would be very useful for restoration research.

## 5. Conclusions

This study provided compelling evidence for stress recovery effects of autumn campus street trees in virtual visual stimulation on graduate students. A brief visual experience increased R-R intervals, alpha and beta brainwaves, reduced anxiety levels, and enhanced perceived restorativeness. The street trees *G. biloba* and *S. japonica* exhibited more pronounced impacts on stress recovery, whereas the effects of *P. acerifolia* and *K. paniculata* were relatively weak. These findings demonstrated the active roles of autumn campus street trees in human stress release.

## Figures and Tables

**Table 1:** The morphological characteristics of street trees.

Street Trees Species	Morphological Characteristics
*Sophora japonica*	A deciduous tree, up to 25 m, trunk straight, crown wide, bark gray-brown and longitudinally striate; pinnately compound, dark green leaves in summer and yellow withered in late autumn.
*Ginkgo biloba*	A deciduous tree, up to 20 m, trunk straight, crown conical, bark grayish brown and longitudinally fissured; fan-shaped, pale green leaves in summer and golden yellow in autumn.
*Platanus acerifolia*	A deciduous tree, up to 25 m, trunk straight, crown broadly ovoid, smooth bark light gray and mottled white; broadly ovate, (3 or) 5-lobed, dark green leaves in summer and yellow-brown in autumn.
*Koelreuteria paniculata*	A deciduous tree, up to 20 m, crown broad and dome-shaped, bark grayish brown to black; pinnately compound, bright green leaves in summer and yellow in autumn; the fruit is orange to pink and reddish-brown, papery seed capsules which somewhat resemble Chinese lanterns in autumn.

**Table 2:** Electroencephalography (EEG) brainwaves and corresponding brain state.

Brainwaves	Frequencies (Hz)	Brain State
Low alpha	8–10	A sense of security to the surrounding environment, slack, unguarded
High alpha	10–12	No stress and anxiety, relaxed but wakeful
Low beta	12–20	Focused on mental activity
High beta	20–30	Excited, alert, attentive

**Table 3:** Comparison of mean values of R-R intervals and brainwaves before and after the Trier Social Stress Test (TSST).

Parameter	Pre	Post	*t*	*p*	Cohen’s *d*
R-R interval	0.828 ± 0.012	0.712 ± 0.010	15.161	0.000 **	1.029
Low alpha	26,078.684 ± 903.091	21,963.362 ± 1488.589	2.718	0.008 **	0.345
High alpha	30,033.950 ± 2079.774	19,874.067 ± 666.362	4.850	0.000 **	0.679
Low beta	9148.347 ± 684.498	15,835.987 ± 542.500	−10.194	0.000 **	1.117
High beta	7727.177 ± 508.102	14,721.790 ± 565.273	−11.479	0.000 **	1.342

*N* = 150, mean ± SEM. ** *p* < 0.01, paired-sample *t*-test.

**Table 4:** Changes in R-R intervals from the stress stage to the recovery stage.

Scenes	Mean Increase	SEM	95% C.I.		*t*	*p*	Cohen’s *d*
			Lower	Upper			
*Sophora japonica*	0.090	0.017	0.055	0.126	5.293	0.000 **	0.918
*Ginkgo biloba*	0.074	0.016	0.041	0.107	4.715	0.000 **	0.696
*Platanus acerifolia*	0.051	0.018	0.017	0.085	3.380	0.007 **	0.627
*Koelreuteria paniculata*	0.044	0.017	0.007	0.081	2.550	0.022 *	0.407
Indoor environment	0.015	0.010	−0.005	0.035	1.550	0.136	0.150

*N* = 30, mean ± SEM. * *p* < 0.05, ** *p* < 0.01, paired sample *t*-test.
